# Why do platinum catalysts show diverse electrocatalytic performance?

**DOI:** 10.1016/j.fmre.2022.03.017

**Published:** 2022-04-12

**Authors:** Qiangmin Yu, Zhiyuan Zhang, Heming Liu, Xin Kang, Shiyu Ge, Shaohai Li, Lin Gan, Bilu Liu

**Affiliations:** Shenzhen Geim Graphene Center, Tsinghua-Berkeley Shenzhen Institute & Institute of Materials Research, Shenzhen International Graduate School, Tsinghua University, Shenzhen 518055, China

**Keywords:** Platinum, Electrocatalyst, Overpotential, Solution resistance, Loading quantity, Microstructure, Catalyst area, Evaluation criteria

## Abstract

As one of the best electrocatalysts for the hydrogen evolution reaction, platinum catalysts are a benchmark for the performance evaluation of new catalysts. However, platinum catalysts reported in the literature show diverse electrocatalytic performances, resulting in the lack of a common reference standard. In this study, we investigated several factors that affect the performance of platinum catalysts by performing experimental measurements and data processing. These factors included the solution resistance, electrolyte temperature, loading quantity, catalyst microstructure, and normalization method of the current density. Finally, we recommended criteria for the performance evaluation of electrocatalysts.

## Graphical Abstract

**Factors that affect the evaluation of electrocatalyst performance**. These factors include the intrinsic activity, resistance effect, environmental effect, loading quantity, catalyst microstructure, and evaluation method.

**Figure:**
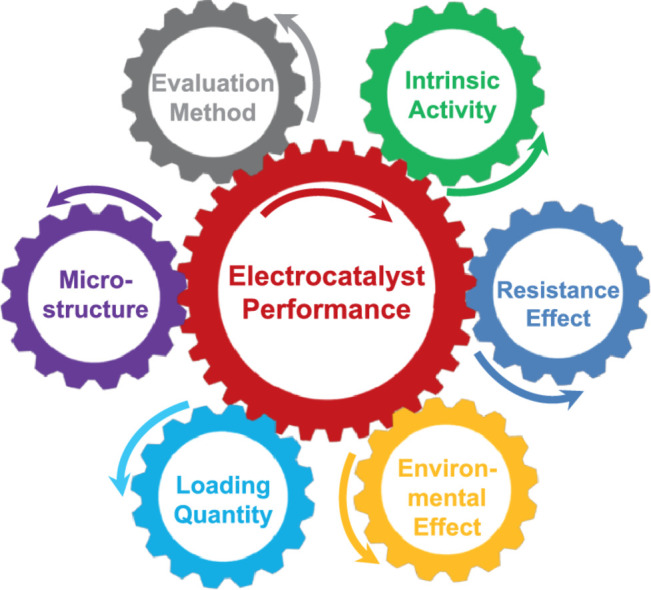

## 1 Introduction

Electrochemical reactions such as the hydrogen evolution reaction (HER), oxygen evolution reaction (OER), oxygen reduction reaction, and carbon dioxide reduction reaction, are playing increasingly important roles in energy conversion devices for sustainable energy technologies and carbon neutrality [1], [2], [3], [4], [5]. For these electrochemical reactions, high-performance electrocatalysts are important to increase the energy conversion efficiency and lower the cost of energy consumption [6], [7], [8], [9], [10]. Thus, scientists have developed various high-performance electrocatalysts to accelerate the reaction rate. In most studies, as-synthesized electrocatalysts exhibited excellent catalytic performance under respective experimental conditions. However, the experimental conditions and performance evaluation methods varied in these studies, making an accurate comparison between the performances of the reported electrocatalysts challenging.

**Fig. 1:**
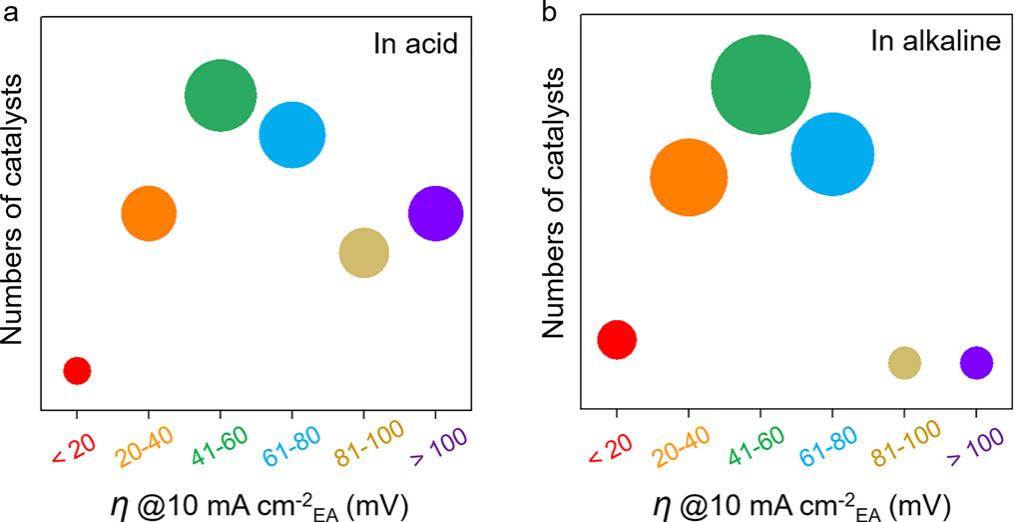
**Summary of HER activity of Pt electrocatalysts reported in the literature**. The overpotentials (*η*) of Pt catalysts at the current density of 10 mA cm^−2^_EA_ (a) in acidic and (b) alkaline electrolytes; the size of the circles represent the number of catalysts in the specified overpotential range. The Pt catalysts include foil, wire, film, and mesh, and the data are collected from the literature. Details are shown in Tables S1 and S2 in the Supplementary Information.

**Fig. 2:**
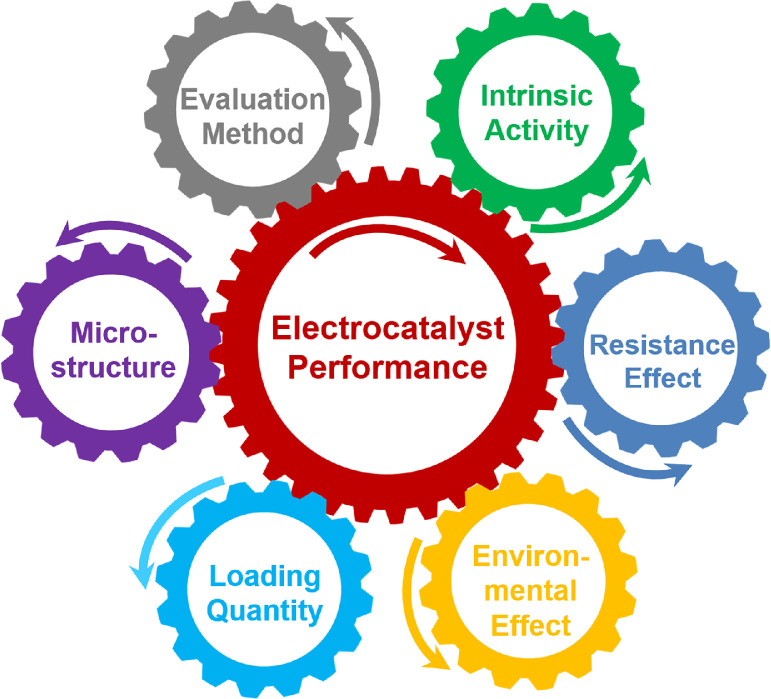
**Factors that affect the evaluation of electrocatalyst performance**.

Overpotential (*η*), the additional potential to drive the reaction to reach a specific current density, is widely used as the primary parameter to evaluate the activity of electrocatalysts [11], [12], [13]. Electrochemical measurements are typically performed in a three-electrode system, and *η* at a current density of 10 mA cm^−2^ (defined as *η* @10 mA cm^−2^ hereafter) is used to describe the activity of electrocatalysts in both the HER and OER [14, 15]. However, the reported *η* @10 mA cm^−2^ shows different results for even the most commonly used reference catalysts, namely, platinum (Pt) catalysts. In Fig. 1, we show *η* @10 mA cm^−2^ for the HER in both acidic (Fig. 1a) and alkaline (Fig. 1b) electrolytes and find that the values of *η* @10 mA cm^−2^ for different Pt catalysts, such as foil, wire, film, or mesh, exhibit a wide range from 20 to 100 mV and sometimes even larger than 100 mV. Although these values are obtained by normalizing the projected electrode area (EA) of the Pt catalysts, the varying morphologies of these Pt catalysts result in different results (Tables S1and S2). In addition, the testing conditions and catalyst features, such as the size of the electrolyzer, type and temperature of the electrolyte, and loading quantity of the catalysts, varied in the previous studies. The *η* of the electrocatalyst changes with not only the catalyst microstructure [16, 17], but also resistances such as solution resistance (*R*_s_) and charge transfer resistance (*R*_ct_) [18, 19]. Furthermore, the working environment, such as the electrolyte temperature, can affect the thermodynamics of the electrocatalyst, resulting in a change in *η* [20], [21], [22]. Fig. 2 presents several factors that may affect the performance of electrocatalysts, including the intrinsic activity, resistance effect, environmental effect, loading quantity, catalyst microstructure, and evaluation method. Therefore, to maintain sound progress in the electrocatalytic field, a standard methodology for measuring and analyzing the catalytic performance of electrocatalysts is urgently required.

Here, we study the underlying reasons for the diverse performances of Pt catalysts and aim to provide evaluation criteria for reliable electrochemical measurements. We focus on the following two aspects: The first aspect is related to the experimental measurements, including: (i) the distance between the reference electrode (RE) and working electrode (WE), which represents *R*_s_; (ii) the temperature of the electrolyte, and (iii) the loading quantity and microstructure of the catalysts. The second aspect is related to data processing, that is, the method for determining and normalizing the area of the catalysts. Finally, we propose a set of standardized testing protocols and data processing methods to evaluate the performance of the electrocatalyst.

## 2 Experimental section

Pt foil (purity: 99.5%), Pt/C with different mass ratios of Pt, and Pt black were purchased from Alfa Aesar. IrO_2_ was purchased from Aladdin. Tantalum disulfides were synthesized using chemical vapor deposition method, as previously reported [23]. All electrochemical measurements were conducted on a VMP-300 electrochemical station (Bio-Logic, France) with a standard three-electrode system in a 0.5 M H_2_SO_4_ or a 1.0 M KOH electrolyte. A graphite rod was used as the counter electrode (CE), a saturated Ag/AgCl electrode (in acidic electrolyte) or an Hg/HgO (in alkaline electrolyte) as the RE, and the platinum-based catalysts as the WE for the HER and commercial IrO_2_ for the OER tests. Prior to the electrochemical tests, the electrolyte was purged with N_2_ gas (99.999%) for 30 min. All reported potentials were calibrated using a reversible hydrogen electrode (RHE). The calibration of the saturated Ag/AgCl electrode was performed in a hydrogen-saturated electrolyte made of 0.5 M H_2_SO_4_ with Pt foil as both the WE and CE. The Hg/HgO electrode was calibrated in a hydrogen-saturated electrolyte made of 1.0 KOH with Pt foil as both the WE and CE. Linear sweep voltammetry (LSV) tests were performed at a scan rate of 0.5 mV s^−1^. The average of the two potentials at which the current crossed zero was considered the thermodynamic potential for the hydrogen electrode reaction. The calibration in a 0.5 M H_2_SO_4_ electrolyte was based on the following equation: *E*_(RHE)_ = *E* (Ag/AgCl) + 0.0591pH + 0.205. The calibration in a 1.0 KOH electrolyte was based on the following equation: *E*_(RHE)_ = *E* (Hg/HgO) + 0.0591pH + 0.097. The HER activity of the different samples was evaluated based on LSV with a scan rate of 2 mV s^−1^. Nyquist plots were obtained at an overpotential of 30 mV, and the sweeping frequencies ranged from 1 MHz to 0.1 Hz. The electrochemical surface area (ECSA) of the catalysts was calculated by using the following equation:

(1) $$\begin{array}{c} \text{ECS}A_{\text{Pt}}=C_{\text{dl}}/\left(0.196\text{mF}\;cm^{-2}\right) \end{array}$$

where *C*_dl_ is the capacitance of the electrochemical double-layer and the coefficient 0.196 mF cm^−2^ is the value of the specific capacitance on polycrystalline Pt.

The hydrogen desorption area (HDA) of the catalyst was calculated by using the following equation:

(2) $$\text{HD}A_{\text{Pt}}=Q_{H}/\left(210\;\mu C\;cm^{-2}\right)$$

where *Q*_H_ is the corrected charge for hydrogen desorption and the coefficient 210 µC cm^−2^ is the value of the hydrogen desorption charge density on polycrystalline Pt.

## 3 Results and discussion

**Fig. 3:**
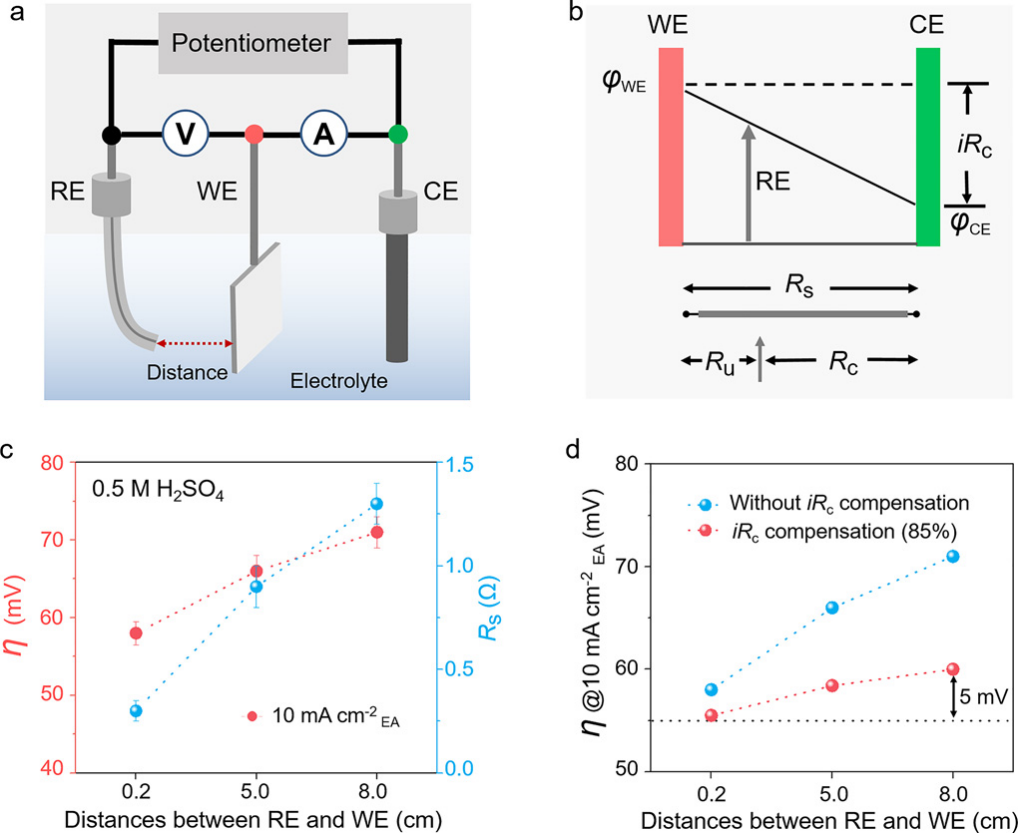
**Catalytic activity of Pt foil *vs.* solution resistance**. (a) Schematic of a three-electrode electrochemical system. (b) Schematic of a three-electrode cell with different *R*_s_ tuned by the distances between the reference electrode (RE) and working electrode (WE); *R*_s_, *R*_c_, *R*_u_ represent solution, compensated, and uncompensated resistances, respectively. (c) The overpotentials of Pt foil at the current density of 10 mA cm^−2^_EA_ and *R*_s_ at different distances between the RE and WE in a 0.5 M H_2_SO_4_ electrolyte. (d) The overpotentials of Pt foil at the current density of 10 mA cm^−2^_EA_ with or without the *iR*_c_ compensation (85%).

To investigate the origin of the varied performances of the Pt catalysts, we used commercial Pt catalysts in HER tests. First, we explored the effect of resistance on catalytic performance using Pt foil (Figs. S1 and S2) as the WE in a three-electrode cell, which consists of two circuits (Fig. 3a). The polarization circuit between the WE and CE monitors the charge transfer and polarization current, and the measuring circuit between the RE and WE controls the potential and measures the electrochemical reaction process of the WE [24]. A voltage drop between the RE and WE is generated due to the existence of *R*_s_, and the potential can be partially corrected by performing *iR*_c_ compensation (Fig. 3b). *R*_s_ increases with increasing distance between the RE and WE; thus, the potential (between the WE and CE) in the reaction will decrease. The activity of the Pt catalysts normalized by EA at a current density of 10 mA cm^−2^ (*η* @10 mA cm^−2^_EA_) was first evaluated by varying the distances between the RE and WE (Fig. S3a). The results show that the *η* @10 mA cm^−2^_EA_ of Pt catalysts increased from 58 to 71 mV (without *iR*_c_ compensation) when the distances between the RE and WE increased from 0.2 to 8.0 cm (Figs. 3c and S3b; in 0.5 M H_2_SO_4_ electrolyte). Electrochemical impedance spectroscopy (EIS) results show that the *R*_s_ also increased from 0.3 to 1.3 Ω under these conditions (Fig. 3c). Similar results are obtained in 1.0 M KOH electrolyte, where *η* @10 mA cm^−2^_EA_ of Pt catalysts increases from 63 to 72 mV (without *iR*_c_ compensation) and the *R*_s_ also increases from 0.3 to 1.2 Ω when the distances between the WE and RE increased from 0.2 to 8.0 cm (Figs. S3c and S3d). Such increases in overpotential occur when the distances between the RE and WE are increased owing to the extra voltage drop caused by the increase in R*_s_*. Although we calculated *η* @10 mA cm^−2^_EA_ with *iR*_c_ compensations (85%) (Fig. 3d), the voltage loss remained. Completely eliminating the resistance effect is difficult because uncompensated resistance (*R*_u_) still remains in the electrochemical cell. Resistance effects are common in the electrochemical systems, regardless of the catalytic material or reaction. In addition to the Pt foil for HER, we investigated the effect of resistance on the activity of different catalyst materials (for example, tantalum disulfides for the HER; Fig. S4) and reactions (for example, iridium dioxides for the OER; Fig. S5). Both cases show that the *η* @10 mA cm^−2^_EA_ of the catalyst and the *R_s_* increase when the distances between the RE and WE increase from 0.2 to 8.0 cm. These results reconfirm the influence of the resistance effect on the performance of the electrocatalysts. The actual activity of the catalyst can only be accurately measured by minimizing the resistance effect as much as possible.

**Fig. 4:**
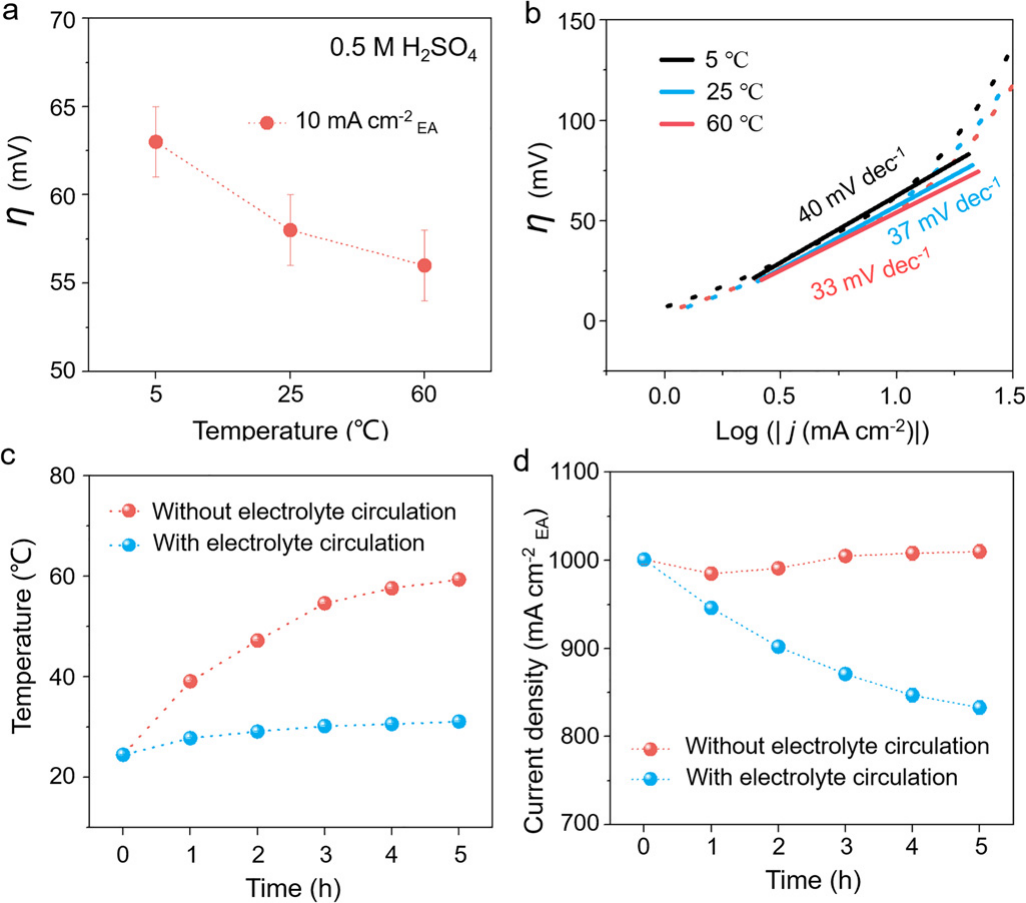
**Effect of electrolyte temperature on catalytic performance**. (a) Overpotentials (*η*) of Pt foil catalysts at current density of 10 mA cm^−2^ normalized by electrode area (EA) at different electrolyte temperatures of 5, 25, and 60 °C in a 0.5 M H_2_SO_4_ electrolyte. (b) Tafel slopes of Pt foil catalysts at different electrolyte temperatures. (c) Electrolyte temperature versus time with and without electrolyte circulation at 1,000 mA cm^−2^_EA_. (d) *i-t* curves of Pt foil catalysts with and without electrolyte circulation.

Next, we studied the environmental effects on the performance of the catalysts by changing the electrolyte temperature. Three electrochemical systems were designed with different electrolyte temperatures (5, 25, and 60 °C) to investigate the HER activity of the Pt foil catalyst. We found that the *η* gradually decreased as the electrolyte temperature increased (Figs. 4a and S6). The corresponding Tafel slope also decreased from 40 to 33 mV dec^−1^ as the electrolyte temperature increased from 5 to 60 °C (Fig. 4b). These results are consistent with the Arrhenius equation,$\;k=A\text{exp}(-E_{a}/RT)$, where *k* is the rate constant of the reaction, *A* is the pre-exponential factor, *E*_a_ is the activation energy, and *T* is the temperature. Note that the electrolyte temperature may increase significantly owing to the heat effect caused by ohmic heating under large-current-density operation. Our results show that the electrolyte temperature does not change significantly when the operating current density is less than 100 mA cm^−2^, whereas it increases sharply when the operating current density is greater than 500 mA cm^−2^ (Fig. S7). For example, the electrolyte temperature increases from 25.0 °C to 69.1 °C at 2,000 mA cm^−2^_EA_ after 5 h of operation. Therefore, the loss of performance may be offset by the heat effect during the stability test at large current densities, resulting in a false-positive result. Here, we investigate the effect of heat on the electrocatalyst stability by controlling the electrolyte temperature with or without electrolyte circulation. As shown in Fig. 4c, the electrolyte temperature gradually increases during the stability testing at 1,000 mA cm^−2^_EA_ without electrolyte circulation, whereas the temperature remains constant when we use the circulated electrolyte. As a result, after a 5 h stability test, the current density shows negligible decay without circulation, which is misleading, whereas it decreases by 18% with electrolyte circulation, which is expected. The degradation of the catalyst performance was due to the protonation of the active sites on the Pt surface as the reaction progressed (Fig. 4d). The thermodynamics of the electrocatalyst without electrolyte circulation will improve when the working temperature increases owing to the heat effect, which compensates for the decrease in the current density over long-term operation. This phenomenon usually causes a misleading evaluation of the electrocatalyst stability, especially under large-current-density operation. Therefore, care must be taken to eliminate the temperature effect when evaluating the large-current-density performance of electrocatalysts.

**Fig. 5:**
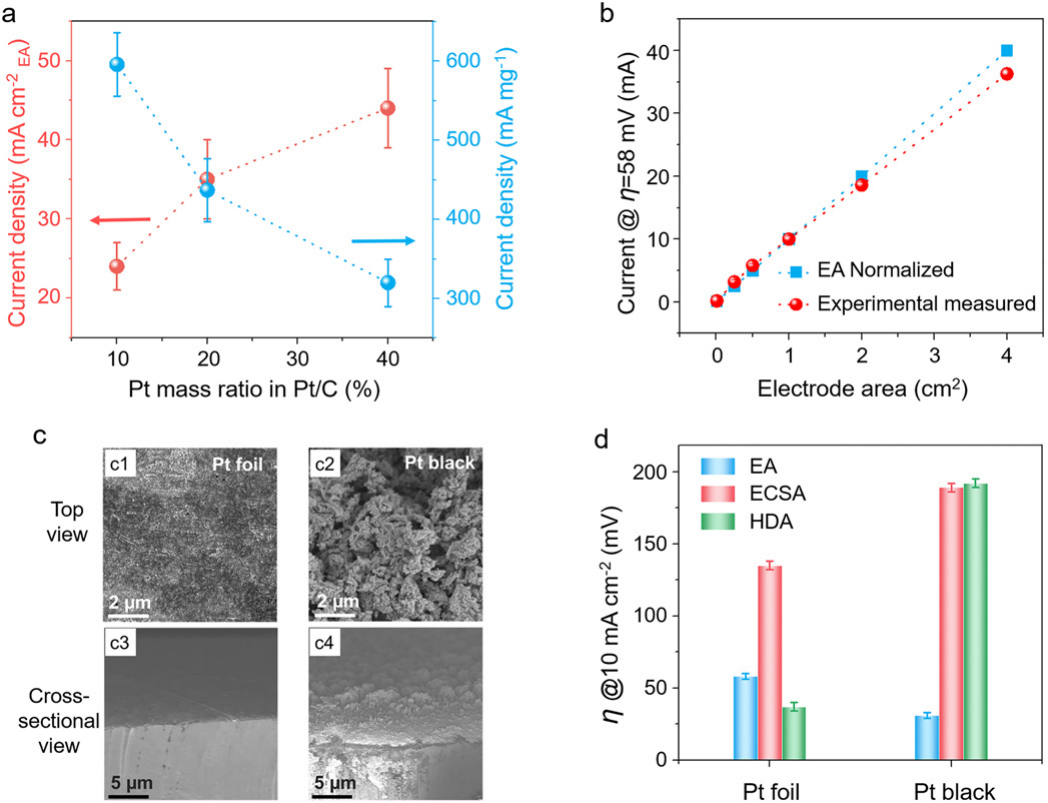
**Catalytic activity of Pt catalysts based on the EA, loading quantity, size, and different methods to normalize catalyst areas**. (a) Current density of different mass ratios of Pt (10%, 20%, and 40%) of Pt/C catalysts in a 0.5 M H_2_SO_4_ electrolyte calculated by both EA and loading quantity; the loading quantities of Pt are 8 µg, 16 µg, and 32 µg. (b) Measured and EA-normalized current densities of Pt catalysts at a constant overpotential of 58 mV; Pt catalysts with different sizes (i.e., EA) are used during the experiments. (c) Scanning electron microscopy images of Pt foil (left panel) and Pt black (right panel) from top and cross-sectional views. (d) Overpotentials of Pt foil and Pt black at 10 mA cm^−2^ (*η* @ 10 mA cm^−2^) with different methods to normalize catalyst areas, including the EA, ECSA, and HDA.

We investigated the effects of the loading quantity and microstructure of the Pt catalysts on their performance. In the case of noble metal catalysts, such as Pt, different mass ratios of Pt in commercial Pt/C catalysts have also been used to compare their catalytic activities to those in the literature. We conducted the electrochemical tests based on three different mass ratios of Pt under the same conditions and normalized their activities by using both the projected EA and quantity of Pt (Fig. S8). The results show that the current density calculated from EA increased from 24 to 44 mA cm^−2^ whereas that calculated from the quantity of Pt decreased from 596 to 320 mA mg^−1^ when the mass ratios of Pt in Pt/C catalysts increased from 10% to 40% at a *η* of 50 mV (Fig. 5a). These results indicate that for the same catalyst, the opposite trend in catalytic performance can be observed using different evaluation methods. In addition, we found that even the EA-normalized current differs depending on the electrode size (Figs. 5b and S9). Electrodes with different sizes (i.e., different EA) may cause different degrees of uneven distribution of electric fields, polarization, and *R*_s_, resulting in different reaction dynamics of the local active sites on the catalysts. To avoid these effects, we recommend that the EA of the CE should be twice larger than that of the WE in a three-electrode electrochemical cell. We also found that the catalytic performance showed significant differences, even though the electrodes had the same geometric EA. Two catalysts were used: Pt black (Pt nanoparticles loaded on a Pt foil) and Pt foil. As shown in Fig. 5c, Pt black has a porous structure, whereas the Pt foil is flat. Therefore, Pt black has a much larger surface area than flat Pt foil, indicating that a large number of active sites can be exposed in the electrolyte in the former case. The catalytic activities of Pt black and Pt foil were investigated based on different types of normalized areas, including EA, ECSA (calculated by Eq. 1), and HDA (calculated by Eq. 2) (Figs. 5d and S10). The results show that Pt black only needs a *η* of 31 mV to reach 10 mA cm^−2^ normalized by EA, which is smaller than that of the Pt foil (57 mV). However, the *η* @ 10 mA cm^−2^ values of Pt black normalized by ECSA and HDA (189 mV and 192 mV, respectively) are larger than those of Pt foil (135 mV and 37 mV, respectively). There are two possible reasons for this result. First, the surface average coordination number of Pt black is lower than that of Pt foil, resulting in worse activity of Pt black than Pt foil. Second, the proportion of active sites involved in the HER of Pt black is smaller than that of Pt foil owing to the limited gas diffusion and mass transfer in Pt black. Therefore, the evaluation of the catalytic activity differs when different methods are used to normalize the catalyst areas.

## 4 Conclusion and Outlook

We found that the performance of Pt electrocatalysts varied with different experimental conditions and evaluation methods, including the resistance effect, electrolyte temperature, loading quantity, catalyst microstructure, and methods to normalize the catalyst area. We advocate the establishment of testing and evaluation criteria to examine the performance of the electrocatalysts more accurately and reliably. The following are some proposed guidelines for accurately evaluating the performance of electrocatalysts.

(a) **Provide a checklist of experimental details and evaluation methods**. The detailed measurement conditions and evaluation method of the electrocatalysts should be described in full (Table 1). First, to fairly compare the testing results and evaluate the practicality of the electrocatalyst, the electrochemical cell should maintain the same conditions during the testing process and be as consistent with industrial standards as possible. Second, criteria such as environmental temperature, pH value, pressure, testing parameters, and evaluation methods should be presented in detail in the electrocatalytic performance statement. Third, reasonable use of RE and CE is critical for performance evaluation: we recommend that for the RE using a saturated calomel electrode or hydrogen electrode in acidic electrolytes and mercuric oxide electrodes in alkaline electrolytes. In addition, platinum- and graphite-based electrodes are not suitable for use as OER electrodes in acidic electrolytes because the dissolved matter from the CE may migrate to the surface of the WE and affect its catalytic activity.

**Table 1:** **Checklist for the evaluation criteria of electrocatalytic performance**.

(b) **Minimize the resistance effect on performance evaluation**. The resistance effect causes issues in electrolysis testing, which would make the measurement of the potential inaccurate. We suggest that researchers shorten the distance between the RE and WE in an electrochemical cell, and use the Luggin-Haber capillary to lower *R*_s_. In addition, *iR* compensation is required to minimize the resistance effect of the electrolyte on the activity evaluation. Furthermore, the interference of the thermal effect should be avoided by using a circulated electrolyte, which is necessary especially when operating at a large current density over a long time.

(c) **Set normalization standards**. The selection of the catalyst area for the normalization of the current density generally depends on the type and microstructure of the catalysts. Regarding non-noble metal electrocatalysts, we recommend using EA to normalize the current density for flat catalysts and using ECSA or HDA to normalize the current density for porous catalysts. For noble metal electrocatalysts, the quantity of noble metal catalysts used is of concern because they share a noticeable percentage of the cost in the energy conversion system. In this case, mass activity should be used, which is generally suitable for noble metal electrocatalysts because of their high cost and scarcity.

(d) **Define the performance evaluation method**. The evaluation methods for catalytic performance vary because of the different types of catalytic materials and their different purposes. Generally, evaluation methods can be divided into two categories; one is the evaluation of the intrinsic catalytic activity, and the other is the evaluation of the overall catalyst performance. In the former case, focusing on fundamental studies, it is necessary to calculate the number of active sites. For the latter case, which is more application-oriented, the loading quantity and cost of the catalyst should be considered, especially when using a large quantity of noble metal catalysts.

Establishing a standardized evaluation system for catalytic activity is a prerequisite for the rapid development and large-scale implementation of electrocatalytic energy conversion technologies. Moreover, the atomic structure of electrocatalysts and active sites must be identified by employing theoretical and in-situ experimental approaches to understand catalyst activity. We believe that on the basis of these, advances in new catalytic materials can be evaluated accurately and reliably.

## Declaration of competing interests

The authors declare that they have no conflicts of interest in this work.

## Appendix Supplementary materials

Image, application 1

Image, application 2

Image, application 3
