# Supplementary material for: Why do platinum catalysts show diverse electrocatalytic performance?
Source: Fundam Res. 2022 Apr 12;3(5):804–8. doi: 10.1016/j.fmre.2022.03.017 (PMC11197565; doi:10.1016/j.fmre.2022.03.017)
Supplement: Supplementary file 1 [file mmc1.docx]

**Supplementary Information for *Fundamental Research***

**Why Do Platinum Catalysts Show Diverse Electrocatalytic Performance?**

Qiangmin Yu, Zhiyuan Zhang, Heming Liu, Xin Kang, Shiyu Ge, Shaohai Li, Lin Gan, and Bilu Liu*

Shenzhen Geim Graphene Center, Tsinghua-Berkeley Shenzhen Institute & Institute of Materials Research, Shenzhen International Graduate School, Tsinghua University, Shenzhen 518055, P. R. China

**Corresponding author:**

Bilu Liu, e-mail: [bilu.liu@sz.tsinghua.edu.cn](mailto:bilu.liu@sz.tsinghua.edu.cn)

**Table S1**. A collection of HER activities of Pt catalysts in acidic electrolyte reported in literature.

| η _(10 mA cm_^-2^_)_  (mV) | IR correction | Electrolyte | Scan rate (mV s^-1^) | Pt materials | References |
| --- | --- | --- | --- | --- | --- |
| 11 | N/A | 0.5 M H_2_SO_4_ | 5 | Pt Microelectrode | R1 |
| 18 | N/A | 0.5 M H_2_SO_4_ | N/A | Pt | R2 |
| 33 | N/A | 0.5 M H_2_SO_4_ | N/A | Pt film | R3 |
| 32 | N/A | 0.5 M H_2_SO_4_ | N/A | Wire | R4 |
| 32 | Yes | 0.5 M H_2_SO_4_ | 2 | Wire | R5 |
| 35 | N/A | 0.5 M H_2_SO_4_ | N/A | Plate | R6 |
| 27 | Yes | 1 M H_2_SO_4_ | 1 | Foil | R7 |
| 20 | N/A | 0.5 M H_2_SO_4_ | N/A | Wire | R8 |
| 30 | N/A | 0.5 M H_2_SO_4_ | 0.5 | Wire | R9 |
| 31 | N/A | 0.5 M H_2_SO_4_ | N/A | Plate | R10 |
| 40 | Yes | 0.1 M HClO_4_ | 1 | Foil | R11 |
| 54 | N/A | 0.5 M H_2_SO_4_ | 5 | Microelectrode | R12 |
| 50 | N/A | 0.5 M H_2_SO_4_ | N/A | Foil | R5 |
| 43 | Yes | 0.5 M H_2_SO_4_ | 5 | Wire | R13 |
| 56 | Yes | 0.5 M H_2_SO_4_ | 2 | Pt | R14 |
| 50 | N/A | 0.5 M H_2_SO_4_ | 5 | Sheet | R15 |
| 59 | N/A | H_2_SO_4_ (pH=2) | N/A | Pt | R16 |
| 44 | N/A | 0.5 M H_2_SO_4_ | 5 | Pt | R17 |
| 60 | N/A | 0.5 M H_2_SO_4_ | 5 | Pt | R18 |
| 52 | Yes | 0.1 M HClO_4_ | 10 | Pt | R19 |
| 48 | Yes | 0.5 M H_2_SO_4_ | 20 | Foil | R20 |
| 47 | Yes | 0.5 M H_2_SO_4_ | 2 | Pt | R21 |
| 53 | Yes | 0.5 M H_2_SO_4_ | N/A | Pt | R22 |
| 55 | N/A | 0.5 M H_2_SO_4_ | 5 | Foil | R23 |
| 65 | N/A | 0.5 M H_2_SO_4_ | 5 | Pt | R24 |
| 73 | N/A | 0.5 M H_2_SO_4_ | 10 | Pt | R25 |
| 79 | Yes | 0.5 M H_2_SO_4_ | 5 | Foil | R26 |
| 64 | Yes | 0.5 M H_2_SO_4_ | 5 | Wire | R27 |
| 65 | N/A | 0.5 M H_2_SO_4_ | 1 | Plate | R28 |
| 76 | N/A | 0.5 M H_2_SO_4_ | 2 | Pt | R29 |
| 66 | N/A | 0.5 M H_2_SO_4_ | 5 | Pt | R30 |
| 68 | Yes | 0.5 M H_2_SO_4_ | 1 | Pt | R31 |
| 64 | N/A | 0.5 M H_2_SO_4_ | 2 | Pt | R32 |
| 70 | No | 0.5 M H_2_SO_4_ | 5 | Pt | R33 |
| 100 | N/A | 0.5 M H_2_SO_4_ | 5 | Foil | R34 |
| 150 | N/A | 0.5 M H_2_SO_4_ | 10 | Bulk Pt | R35 |

**Table S2**. A summary of HER activities of Pt catalysts in alkaline electrolyte reported in literature.

| η _(10 mA cm_^-2^_)_  (mV) | IR correction | Electrolyte | Scan rate  (mV s^-1^) | Pt materials | References |
| --- | --- | --- | --- | --- | --- |
| 17 | Yes | 1 M KOH | 5 | Wire | R36 |
| 30 | Yes | 1 M KOH | 2 | Wire | R37 |
| 28 | Yes | 1 M KOH | 2 | Wire | R38 |
| 30 | Yes | 1 M KOH | 2 | Wire | R39 |
| 32 | N/A | 1 M KOH | 5 | Pt | R40 |
| 35 | Yes | 1 M KOH | 5 | Sheet | R41 |
| 45 | N/A | 1 M KOH | 50 | Wire | R42 |
| 57 | N/A | 1 M KOH | 10 | Mesh | R43 |
| 59 | N/A | 1 M KOH | 1 | Wire | R44 |
| 51 | Yes | 1 M KOH | 10 | Pt | R45 |
| 60 | Yes | 1 M KOH | 5 | Foil | R46 |
| 44 | N/A | 1 M KOH | 10 | Pt | R47 |
| 45 | Yes | 1 M KOH | 2 | Plate | R10 |
| 50 | Yes | 1 M KOH | 2 | Mesh | R48 |
| 71 | No | 1 M KOH | 1 | Foil | R49 |
| 78 | No | 1 M KOH | 5 | Foil | R50 |
| 70 | Yes | 1 M KOH | 2 | Foil | R51 |
| 74 | N/A | 1 M KOH | 5 | Foil | R52 |
| 68 | Yes | 1 M KOH | 5 | Plate | R53 |
| 78 | No | 1 M KOH | 0.5 | Film | R54 |
| 70 | Yes | 1 M KOH | 5 | Sheet | R15 |
| 83 | Yes | 1 M KOH | 5 | Foil | R55 |
| 84 | Yes | 1 M KOH | 5 | Pt | R56 |
| 85 | N/A | 1 M KOH | 2 | Pt | R8 |
| 83 | Yes | 1 M KOH | 2 | Wire | R57 |
| 102 | Yes | 0.1 M KOH | 5 | Wire | R58 |
| 120 | N/A | 1 M KOH | N/A | Disk | R59 |
| 181 | N/A | 0.1 M KOH | 5 | Foil | R60 |
| 104 | Yes | 0.1 M KOH | 10 | Wire | R19 |
| 128 | Yes | 1 M KOH | 5 | Pt | R61 |

**Figure S1**. SEM images of (a) Pt foil and (b) Pt black used in this work. The surface of Pt foil shows flat and the surface of Pt black shows many nanoparticles, which suggests that the numbers of active sites on the surface of Pt black are more than that of Pt foil.

**Figure S2**. Spectroscopy characterization of the Pt foil and Pt black used in this work. The XRD patterns of (a) Pt foil and (b) Pt black. The Pt 4*f* XPS spectra of (c) Pt foil and (d) Pt black. XRD and XPS results show that the Pt foil and Pt black have the same crystal structure with different amount of adsorbed oxygen. This is because there are many Pt nanoparticles in Pt black, which makes it adsorb more oxygen than that of Pt foil.

**Figure S3**. (a) The schematic of three-electrode cell with different distances between RE and WE. (b) HER polarization curves of Pt foil with different electrode distances between RE and WE in a 0.5 M H_2_SO_4_ electrolyte. (c) HER polarization curves of Pt foil with different electrode distances between RE and WE in a 1.0 M KOH electrolyte and (d) corresponding overpotentials at the current density of 10 mA cm^-2^_EA_ and resistance of solution (R*_s_*).

Note that the R*_s_* has a linear relationship with the distances between RE and WE, the relationship is as follows:

$$R_{s}= \rho\frac{L}{S}$$

Where the ρ is the resistivity of solution, the *L* is the distances between RE and WE, and the S is the electrode area immersed in solution.

**Figure S4**. (a) HER polarization curves of TaS_2_ electrocatalyst with different electrode distances between RE and WE in a 0.5 M H_2_SO_4_ electrolyte. (b) Corresponding overpotentials at the current density of 10 mA cm^-2^ _EA_ and R*_s_* with different distances between RE and WE.

**Figure S5**. (a) OER polarization curves of IrO_2_ electrocatalyst with different electrode distances between RE and WE in a 1.0 M KOH electrolyte. (b) Corresponding overpotentials at the current density of 10 mA cm^-2^ _EA_ and R*_s_* with different distances between RE and WE.

**Figure S6**. HER polarization curves of Pt foil with different electrolyte temperatures in a 0.5 M H_2_SO_4_ electrolyte.

**Figure S7.** The electrolyte temperature versus time without electrolyte circulation tested at different operating current densities of 10, 100, 500, 1000, and 2000 mA cm^-2^. The catalyst is Pt foil.

**Figure S8**. HER polarization curves of Pt/C with different Pt quantity loadings in a 0.5 M H_2_SO_4_ electrolyte.

**Figure S9**. HER polarization curves of Pt foil with different electrode areas in a 0.5 M H_2_SO_4_ electrolyte.

**Figure S10**. (a) HER polarization curves of Pt foil and Pt black in a 0.5 M H_2_SO_4_ electrolyte. The capacitive current at 0.5 V against the scan rate and corresponding capacitive of double-layer (C*_dl_*) of (b) Pt foil and (c) Pt black estimated by linear fitting of the plots. The HDA curves of (d) Pt foil and (e) Pt black.

**Supplementary References**

1. Yu, Y.; Nam, G. H.; He, Q.; Wu, X. J.; Zhang, K.; Yang, Z.; Chen, J.; Ma, Q.; Zhao, M.; Liu, Z.; Ran, F. R.; Wang, X.; Li, H.; Huang, X.; Li, B.; Xiong, Q.; Zhang, Q.; Liu, Z.; Gu, L.; Du, Y.; Huang, W.; Zhang, H. High Phase-Purity 1T'-MoS_2_- and 1T'-MoSe_2_-Layered Crystals. *Nat. Chem.* **2018**, *10*, 638-643.

2. Yang, J.; Mohmad, A. R.; Wang, Y.; Fullon, R.; Song, X.; Zhao, F.; Bozkurt, I.; Augustin, M.; Santos, E. J. G.; Shin, H. S.; Zhang, W.; Voiry, D.; Jeong, H. Y.; Chhowalla, M. Ultrahigh-Current-Density Niobium Disulfide Catalysts for Hydrogen Evolution. *Nat. Mater.* **2019**, *18*, 1309-1314.

3. Song, Q.; Xue, Z.; Liu, C.; Qiao, X.; Liu, L.; Huang, C.; Liu, K.; Li, X.; Lu, Z.; Wang, T. General Strategy to Optimize Gas Evolution Reaction via Assembled Striped-Pattern Superlattices. *J. Am. Chem. Soc.* **2020**, *142*, 1857-1863.

4. Zhou, H.; Yu, F.; Liu, Y.; Sun, J.; Zhu, Z.; He, R.; Bao, J.; Goddard, W. A.; Chen, S.; Ren, Z. Outstanding Hydrogen Evolution Reaction Catalyzed by Porous Nickel Diselenide Electrocatalysts. *Energ. Environ. Sci.* **2017**, *10*, 1487-1492.

5. Zhou, H.; Yu, F.; Sun, J.; Zhu, H.; Mishra, I. K.; Chen, S.; Ren, Z. Highly Efficient Hydrogen Evolution from Edge-Oriented WS_2(1-x)_Se_2x_ Particles on Three-Dimensional Porous NiSe_2_ Foam. *Nano Lett.* **2016**, *16*, 7604-7609.

6. Gao, J.; Cheng, Z.; Shao, C.; Zhao, Y.; Zhang, Z.; Qu, L. A 2D Free-Standing Film-Inspired Electrocatalyst for Highly Efficient Hydrogen Production. *J. Mater. Chem. A* **2017**, *5*, 12027-12033.

7. Laursen, A. B.; Patraju, K. R.; Whitaker, M. J.; Retuerto, M.; Sarkar, T.; Yao, N.; Ramanujachary, K. V.; Greenblatt, M.; Dismukes, G. C. Nanocrystalline Ni_5_P_4_: a Hydrogen Evolution Electrocatalyst of Exceptional Efficiency in Both Alkaline and Acidic Media. *Energ. Environ. Sci.* **2015**, *8*, 1027-1034.

8. Zhang, Y.; Gao, L.; Hensen, E. J. M.; Hofmann, J. P. Evaluating the Stability of Co_2_P Electrocatalysts in the Hydrogen Evolution Reaction for Both Acidic and Alkaline Electrolytes. *ACS Energ. Lett.* **2018**, *3*, 1360-1365.

9. Zhou, H.; Yu, F.; Huang, Y.; Sun, J.; Zhu, Z.; Nielsen, R. J.; He, R.; Bao, J.; Goddard, W. A., III; Chen, S.; Ren, Z. Efficient Hydrogen Evolution by Ternary Molybdenum Sulfoselenide Particles on Self-Standing Porous Nickel Diselenide Foam. *Nat. Commun.* **2016**, *7*, 12765.

10. Zhang, X.; Yu, X.; Zhang, L.; Zhou, F.; Liang, Y.; Wang, R. Molybdenum Phosphide/Carbon Nanotube Hybrids as pH-Universal Electrocatalysts for Hydrogen Evolution Reaction. *Adv. Funct. Mater.* **2018**, *28*, 1706523.

11. Zhang, J.; Wang, T.; Liu, P.; Liao, Z.; Liu, S.; Zhuang, X.; Chen, M.; Zschech, E.; Feng, X. Efficient Hydrogen Production on MoNi_4_ Electrocatalysts with Fast Water Dissociation Kinetics. *Nat. Commun.* **2017**, *8*, 15437.

12. Meng, Y.; Xue P.; Pei, W.; Fei C.; Liang H.; Geng, J.; Jun W.; Jefferson, L.; Xu, X.; Xiao, L.; Ji, Y.; Li, M. Field-Effect Tuned Adsorption Dynamics of VSe_2_ Nanosheets for Enhanced Hydrogen Evolution Reaction. *Nano Lett.* **2017**, *17*, 4109–4115.

13. Yuan, J.; Wu, J.; Hardy, W. J.; Loya, P.; Lou, M.; Yang, Y.; Najmaei, S.; Jiang, M.; Qin, F.; Keyshar, K.; Ji, H.; Gao, W.; Bao, J.; Kono, J.; Natelson, D.; Ajayan, P. M.; Lou, J. Facile Synthesis of Single Crystal Vanadium Disulfide Nanosheets by Chemical Vapor Deposition for Efficient Hydrogen Evolution Reaction. *Adv. Mater.* **2015**, *27*, 5605-5609.

14. Jiang, Z.; Zhou, W.; Hong, A.; Guo, M.; Luo, X.; Yuan, C. MoS_2_ Moiré Superlattice for Hydrogen Evolution Reaction. *ACS Energ. Lett.* **2019**, *4*, 2830-2835.

15. Zhang, R.; Wang, X.; Yu, S.; Wen, T.; Zhu, X.; Yang, F.; Sun, X.; Wang, X.; Hu, W. Ternary NiCo_2_ P_x_ Nanowires as pH-Universal Electrocatalysts for Highly Efficient Hydrogen Evolution Reaction. *Adv. Mater.* **2017**, *29*. 1605502.

16. Li, H.; Tsai, C.; Koh, A. L.; Cai, L.; Contryman, A. W.; Fragapane, A. H.; Zhao, J.; Han, H. S.; Manoharan, H. C.; Abild-Pedersen, F.; Norskov, J. K.; Zheng, X. Corrigendum: Activating and Optimizing MoS_2_ Basal Planes for Hydrogen Evolution through the Formation of Strained Sulphur Vacancies. *Nat. Mater.* **2016**, *15*, 364.

17. Wu, M. Y.; Da, P. F.; Zhang, T.; Mao, J.; Liu, H.; Ling, T. Designing Hybrid NiP_2_/NiO Nanorod Arrays for Efficient Alkaline Hydrogen Evolution. *ACS Appl. Mater. Interf.* **2018**, *10*, 17896-17902.

18. Zhou, Y.; Silva, J. L.; Woods, J. M.; Pondick, J. V.; Feng, Q.; Liang, Z.; Liu, W.; Lin, L.; Deng, B.; Brena, B.; Xia, F.; Peng, H.; Liu, Z.; Wang, H.; Araujo, C. M.; Cha, J. J. Revealing the Contribution of Individual Factors to Hydrogen Evolution Reaction Catalytic Activity. *Adv. Mater.* **2018**, *30*, e1706076.

19. Yu, X.; Zhao, J.; Zheng, L.-R.; Tong, Y.; Zhang, M.; Xu, G.; Li, C.; Ma, J.; Shi, G. Hydrogen Evolution Reaction in Alkaline Media: Alpha- or Beta-Nickel Hydroxide on the Surface of Platinum? *ACS Energ. Lett.* **2017**, *3*, 237-244.

20. Zhang, Q.; Jiang, Z.; Tackett, B. M.; Denny, S. R.; Tian, B.; Chen, X.; Wang, B.; Chen, J. G. Trends and Descriptors of Metal-Modified Transition Metal Carbides for Hydrogen Evolution in Alkaline Electrolyte. *ACS Catal.* **2019**, *9*, 2415-2422.

21. Wang, Z. J.; Li, M. X.; Yu, J. H.; Ge, X. B.; Liu, Y. H.; Wang, W. H. Low-Iridium-Content IrNiTa Metallic Glass Films as Intrinsically Active Catalysts for Hydrogen Evolution Reaction. *Adv. Mater.* **2020**, *32*, e1906384.

22. Ramalingam, V.; Varadhan, P.; Fu, H. C.; Kim, H.; Zhang, D.; Chen, S.; Song, L.; Ma, D.; Wang, Y.; Alshareef, H. N.; He, J. H. Heteroatom-Mediated Interactions between Ruthenium Single Atoms and an MXene Support for Efficient Hydrogen Evolution. *Adv. Mater.* **2019**, *31*, e1903841.

23. Li, G.; Chen, Z.; Li, Y.; Zhang, D.; Yang, W.; Liu, Y.; Cao, L. Engineering Substrate Interaction To Improve Hydrogen Evolution Catalysis of Monolayer MoS_2_ Films beyond Pt. *ACS Nano* **2020**, *14*, 1707-1714.

24. Chang, B.; Zhao, G.; Shao, Y.; Zhang, L.; Huang, B.; Wu, Y.; Hao, X. Photo-Enhanced Electrocatalysis of Sea-Urchin Shaped Ni_3_(VO_4_)_2_ for the Hydrogen Evolution Reaction. *J. Mater. Chem. A* **2017**, *5*, 18038-18043.

25. Ito, Y.; Cong, W.; Fujita, T.; Tang, Z.; Chen, M. High Catalytic Activity of Nitrogen and Sulfur Co-Doped Nanoporous Graphene in the Hydrogen Evolution Reaction. *Angew. Chem. Inter. Edit.* **2015**, *54*, 2131-2136.

26. Wang, X.; Tai, G.; Wu, Z.; Hu, T.; Wang, R. Ultrathin Molybdenum Boride Films for Highly Efficient Catalysis of The Hydrogen Evolution Reaction. *J. Mater. Chem. A* **2017**, *5*, 23471-23475.

27. Li, F.; Zhao, X.; Mahmood, J.; Okyay, M. S.; Jung, S. M.; Ahmad, I.; Kim, S. J.; Han, G. F.; Park, N.; Baek, J. B. Macroporous Inverse Opal-like Mo_x_C with Incorporated Mo Vacancies for Significantly Enhanced Hydrogen Evolution. *ACS Nano* **2017**, *11*, 7527-7533.

28. Swaminathan, J.; Subbiah, R.; Singaram, V. Defect-Rich Metallic Titania (TiO_1.23_)—An Efficient Hydrogen Evolution Catalyst for Electrochemical Water Splitting. *ACS Catal.* **2016**, 6, 2222-2229.

29. Chia, X.; Sutrisnoh, N. A. A.; Pumera, M. Tunable Pt–MoS_x_ Hybrid Catalysts for Hydrogen Evolution. *ACS Appl. Mater. Interf.* **2018**, *10*, 8702-8711.

30. Bae, C.; Ho, T. A.; Kim, H.; Lee, S.; Lim, S.; Kim, M.; Yoo, H.; Montero-Moreno, J. M.; Park, J. H.; Shin, H. Bulk Layered Heterojunction as an Efficient Electrocatalyst for Hydrogen Evolution. *Sci. Adv.* **2017**, *3*, 1602215.

31. Huang, J.; Chen, M.; Tang, T.; Liu, W.; Liu, Y. Electroplated Synthesis of Semi-Rigid MoS_2_–rGO–Cu as Efficient Self-Supporting Electrode for Hydrogen Evolution Reaction. *Electrochimica Acta* **2020**, *355*, 136754.

32. Zhou, W.; Chen, M.; Guo, M.; Hong, A.; Yu, T.; Luo, X.; Yuan, C.; Lei, W.; Wang, S. Magnetic Enhancement for Hydrogen Evolution Reaction on Ferromagnetic MoS_2_ Catalyst. *Nano Lett.* **2020**, *20*, 2923-2930.

33. Xu, X. Y.; Dong, X. F.; Bao, Z. J.; Wang, R.; Hu, J. G.; Zeng, H. B. Three Electron Channels toward Two Types of Active Sites in MoS_2_@Pt Nanosheets for Hydrogen Evolution. *J. Mater. Chem. A* **2017**, *5*, 22654-22661.

34. Pang, Q. Q.; Niu, Z. L.; Yi, S. S.; Zhang, S.; Liu, Z. Y.; Yue, X. Z. Hydrogen-Etched Bifunctional Sulfur-Defect-Rich ReS_2_/CC Electrocatalyst for Highly Efficient HER and OER. *Small* **2020**, *16*, e2003007.

35. Guha, A.; Veettil Vineesh, T.; Sekar, A.; Narayanaru, S.; Sahoo, M.; Nayak, S.; Chakraborty, S.; Narayanan, T. N. Mechanistic Insight into Enhanced Hydrogen Evolution Reaction Activity of Ultrathin Hexagonal Boron Nitride-Modified Pt Electrodes. *ACS Catal.* **2018**, *8*, 6636-6644.

36. Yu, L.; Mishra, I. K.; Xie, Y.; Zhou, H.; Sun, J.; Zhou, J.; Ni, Y.; Luo, D.; Yu, F.; Yu, Y.; Chen, S.; Ren, Z. Ternary Ni_2(1-x)_Mo_2x_P Nanowire Arrays toward Efficient and Stable Hydrogen Evolution Electrocatalysis under Large-Current-Density. *Nano Energ.* **2018**, *53*, 492-500.

37. Chen, W.; Mishra, I. K.; Qin, Z.; Yu, L.; Zhou, H.; Sun, J.; Zhang, F.; Chen, S.; Wenya, G. E.; Yu, Y.; Wang, Z. M.; Song, H.-Z.; Ren, Z. Nickel Phosphide Based Hydrogen Producing Catalyst with Low Overpotential and Stability at High Current Density. *Electrochimica Acta* **2019**, *299*, 756-761.

38. Mishra, I. K.; Zhou, H.; Sun, J.; Qin, F.; Dahal, K.; Bao, J.; Chen, S.; Ren, Z. Hierarchical CoP/Ni_5_P_4_/CoP Microsheet Arrays as a Robust pH-Universal Electrocatalyst for Efficient Hydrogen Generation. *Energ. Environ. Sci.* **2018**, *11*, 2246-2252.

39. Yu, L.; Zhou, H.; Sun, J.; Qin, F.; Yu, F.; Bao, J.; Yu, Y.; Chen, S.; Ren, Z. Cu Nanowires Shelled with NiFe Layered Double Hydroxide Nanosheets as Bifunctional Electrocatalysts for Overall Water Splitting. *Energ. Environ. Sci.* **2017**, *10*, 1820-1827.

40. Shao, L.; Sun, H.; Miao, L.; Chen, X.; Han, M.; Sun, J.; Liu, S.; Li, L.; Cheng, F.; Chen, J. Facile preparation of NH_2_-Functionalized Black Phosphorene for The Electrocatalytic Hydrogen Evolution Reaction. *J. Mater. Chem. A* **2018**, *6*, 2494-2499.

41. Yi, X.; He, X.; Yin, F.; Chen, B.; Li, G.; Yin, H. Amorphous Ni–Fe–Se Hollow Nanospheres Electrodeposited on Nickel Foam as a Highly Active and Bifunctional Catalyst for Alkaline Water Splitting. *Dalton Trans.* **2020**. *49*, 6764-6775.

42. Digraskar, R. V.; Mali, S. M.; Tayade, S. B.; Ghule, A. V.; Sathe, B. R. Overall Noble Metal Free Ni and Fe Doped Cu_2_ZnSnS_4_ (CZTS) Bifunctional Electrocatalytic Systems for Enhanced Water Splitting Reactions. *Inter. J. Hydro. Energ.* **2019**, *44*, 8144-8155.

43. Read, C. G.; Callejas, J. F.; Holder, C. F.; Schaak, R. E. General Strategy for the Synthesis of Transition Metal Phosphide Films for Electrocatalytic Hydrogen and Oxygen Evolution. *ACS Appl. Mater. Interf.* **2016**, *8*, 12798-12803.

44. Yu, F.; Zhou, H.; Huang, Y.; Sun, J.; Qin, F.; Bao, J.; Goddard, W. A.; Chen, S.; Ren, Z. High-Performance Bifunctional Porous Non-Noble Metal Phosphide Catalyst for Overall Water Splitting. *Nat. Commun.* **2018**, *9*, 2551.

45. Swesi, A. T.; Masud, J.; Liyanage, W. P. R.; Umapathi, S.; Bohannan, E.; Medvedeva, J.; Nath, M. Textured NiSe_2_ Film: Bifunctional Electrocatalyst for Full Water Splitting at Remarkably Low Overpotential with High Energy Efficiency. *Scientific Reports* **2017**, *7*, 2401.

46. Luo, Y.; Tang, L.; Khan, U.; Yu, Q.; Cheng, H.-M.; Zou, X.; Liu, B. Morphology and Surface Chemistry Engineering toward pH-Universal Catalysts for Hydrogen Evolution at High Current Density. *Nat. Commun.* **2019**, *10*, 269.

47. Lao, J.; Li, D.; Jiang, C.; Luo, C.; Qi, R.; Lin, H.; Huang, R.; Waterhouse, G. I. N.; Peng, H. Synergistic Effect of Cobalt Boride Nanoparticles on MoS_2_ Nanoflowers for a Highly Efficient Hydrogen Evolution Reaction in Alkaline Media. *Nanoscale* **2020**, *12*, 10158-10165.

48. Jiang, S. H.; Zhang, R. Y.; Liu, H. X.; Rao, Y.; Yu, Y. N.; Chen, S.; Yue, Q.; Zhang, Y. N.; Kang, Y. J. Promoting Formation of Oxygen Vacancies in Two-Dimensional Cobalt-Doped Ceria Nanosheets for Efficient Hydrogen Evolution. *J. Am. Chem. Soc.* **2020**, *142*, 6461-6466.

49. Li, G.; Fu, C.; Shi, W.; Jiao, L.; Wu, J.; Yang, Q.; Saha, R.; Kamminga, M. E.; Srivastava, A. K.; Liu, E.; Yazdani, A. N.; Kumar, N.; Zhang, J.; Blake, G. R.; Liu, X.; Fahlman, M.; Wirth, S.; Auffermann, G.; Gooth, J.; Parkin, S.; Madhavan, V.; Feng, X.; Sun, Y.; Felser, C. Dirac Nodal Arc Semimetal PtSn_4_ : An Ideal Platform for Understanding Surface Properties and Catalysis for Hydrogen Evolution. *Angew. Chem. Inter. Edit.* **2019**, *58*, 13107-13112.

50. Qiu, Z.; Tai, C.-W.; Niklasson, G. A.; Edvinsson, T. Direct Observation of Active Catalyst Surface Phases and The Effect of Dynamic Self-Optimization in NiFe-Layered Double Hydroxides for Alkaline Water Splitting. *Energ. Environ. Sci.* **2019**, *12*, 572-581.

51. Wang, P.-c.; Wan, L.; Lin, Y.-q.; Wang, B.-g. NiFe Hydroxide Supported on Hierarchically Porous Nickel Mesh as a High-Performance Bifunctional Electrocatalyst for Water Splitting at Large Current Density. *ChemSusChem* **2019**, *12*, 4038-4045.

52. Yu, X.; Wang, M.; Gong, X.; Guo, Z.; Wang, Z.; Jiao, S. Self-Supporting Porous CoP-Based Films with Phase-Separation Structure for Ultrastable Overall Water Electrolysis at Large Current Density. *Adv. Energ. Mater.* **2018**, *8*, 1802445.

53. Qin, F.; Zhao, Z.; Alam, M. K.; Ni, Y.; Robles-Hernandez, F.; Yu, L.; Chen, S.; Ren, Z.; Wang, Z.; Bao, J. Trimetallic NiFeMo for Overall Electrochemical Water Splitting with a Low Cell Voltage. *ACS Energ. Lett.* **2018**, *3*, 546-554.

54. Luo, Y.; Zhang, S.; Pan, H.; Xiao, S.; Guo, Z.; Tang, L.; Khan, U.; Ding, B.-F.; Li, M.; Cai, Z.; Zhao, Y.; Lv, W.; Feng, Q.; Zou, X.; Lin, J.; Cheng, H.-M.; Liu, B. Unsaturated Single Atoms on Monolayer Transition Metal Dichalcogenides for Ultrafast Hydrogen Evolution. *ACS Nano* **2020**, *14*, 767-776.

55. Liu, K.; Wang, F.; Shifa, T. A.; Wang, Z.; Xu, K.; Zhang, Y.; Cheng, Z.; Zhan, X.; He, J. An Efficient Ternary CoP_2x_Se_2(1−x)_ Nanowire Array for Overall Water Splitting. *Nanoscale* **2017**, *9*, 3995-4001.

56. Zhang, X.-Y.; Guo, B.-Y.; Chen, Q.-W.; Dong, B.; Zhang, J.-Q.; Qin, J.-F.; Xie, J.-Y.; Yang, M.; Wang, L.; Chai, Y.-M.; Liu, C.-G. Ultrafine and Highly-Dispersed Bimetal Ni_2_P/Co_2_P Encapsulated by Hollow N-Doped Carbon Nanospheres for Efficient Hydrogen Evolution. *Inter. J. Hydro. Energ.* **2019**, *44*, 14908-14917.

57. Yu, L.; Zhou, H.; Sun, J.; Qin, F.; Luo, D.; Xie, L.; Yu, F.; Bao, J.; Li, Y.; Yu, Y.; Chen, S.; Ren, Z. Hierarchical Cu@CoFe Layered Double Hydroxide Core-Shell Nanoarchitectures as Bifunctional Electrocatalysts for Efficient Overall Water Splitting. *Nano Energ.* **2017**, *41*, 327-336.

58. Li, F.; Zhao, X.; Mahmood, J.; Okyay, M. S.; Jung, S.-M.; Ahmad, I.; Kim, S.-J.; Han, G.-F.; Park, N.; Baek, J.-B. Macroporous Inverse Opal-Like Mo_x_C with Incorporated Mo Vacancies for Significantly Enhanced Hydrogen Evolution. *ACS Nano* **2017**, *11*, 7527-7533.

59. Ledendecker, M.; Schlott, H.; Antonietti, M.; Meyer, B.; Shalom, M. Experimental and Theoretical Assessment of Ni-Based Binary Compounds for the Hydrogen Evolution Reaction. *Adv. Energ. Mater.* **2017**, *7*, 1601735.

60. Tao, S.; Yang, F.; Schuch, J.; Jaegermann, W.; Kaiser, B. Electrodeposition of Nickel Nanoparticles for the Alkaline Hydrogen Evolution Reaction: Correlating Electrocatalytic Behavior and Chemical Composition. *ChemSusChem* **2018**, *11*, 948-958.

61. Wu, M.-Y.; Da, P.-F.; Zhang, T.; Mao, J.; Liu, H.; Ling, T. Designing Hybrid NiP_2_/NiO Nanorod Arrays for Efficient Alkaline Hydrogen Evolution. *ACS Appl. Mater. Interf.* **2018**, *10*, 17896-17902.
