# Supplementary material for: Why do platinum catalysts show diverse electrocatalytic performance?
Source: Fundam Res. 2022 Apr 12;3(5):804–8. doi: 10.1016/j.fmre.2022.03.017 (PMC11197565; doi:10.1016/j.fmre.2022.03.017)
Supplement: Supplementary file 2 [file mmc2.docx]

| Electrode  performance | Electrochemical  cell | Half cell | Solution resistance | IR compensation | |  | Additional comments |
| --- | --- | --- | --- | --- | --- | --- | --- |
|  |  | Full cell |  | No IR compensation | | |  |
|  | Catalyst morphologies | 2D flat | Loading mass |  | | |  |
|  |  | Rough |  |  | | |  |
|  | Test conditions (temperature, pressure, pH value) | | |  | | |  |
|  | Testing parameters | | | Potential window |  | |  |
|  |  |  |  | Scan rate |  | |  |
|  |  |  |  | Types of CE and RE |  | |  |
|  | Evaluation  methods | Measured  value | Overpotential |  | | |  |
|  |  |  | Current |  | | |  |
|  |  |  | Tafel slope |  | | |  |
|  |  |  | TOF |  | | |  |
|  |  |  | Stability test current and duration |  | | |  |
|  |  | Normalized  value | Area activity  (Non-noble metal) | Electrode area | |  |  |
|  |  |  |  | ECSA area | |  |  |
|  |  |  | Mass activity  (Noble metal) |  | | |  |

- Checklist for the evaluation criteria of electrocatalytic performance
