# Supplementary material for: Why do platinum catalysts show diverse electrocatalytic performance?
Source: Fundam Res. 2022 Apr 12;3(5):804–8. doi: 10.1016/j.fmre.2022.03.017 (PMC11197565; doi:10.1016/j.fmre.2022.03.017)
Supplement: Supplementary file 3 [file mmc3.docx]

**Why Platinum Catalysts Show Diverse Electrocatalytic Performance?**

Qiangmin Yu, Zhiyuan Zhang, Heming Liu, Xin Kang, Shiyu Ge, Shaohai Li, Lin Gan, and Bilu Liu*

Shenzhen Geim Graphene Center, Tsinghua-Berkeley Shenzhen Institute & Institute of Materials Research, Shenzhen International Graduate School, Tsinghua University, Shenzhen 518055, P. R. China

Corresponding Author*

Bilu Liu, e-mail: [bilu.liu@sz.tsinghua.edu.cn](mailto:bilu.liu@sz.tsinghua.edu.cn)

**Abstract**

As one of the best electrocatalysts for hydrogen evolution reaction, platinum catalyst is a benchmark for performance evaluation of new catalysts in the literature. However, platinum catalysts show diverse electrocatalytic performance reported from different literature, resulting in the lack of a standard as a reference for performance evaluation of new catalysts. Here, we have investigated several factors that affect the performance evaluation of platinum catalysts from both experimental measurements and data processing aspects. These factors include the solution resistance, electrolyte temperature, loading quantity, microstructure of catalysts, and normalization method of current density. Finally, recommended criteria for the performance evaluation of electrocatalysts are advocated.

**Keywords:** Platinum, electrocatalyst, overpotential, solution resistance, loading quantity, microstructure, catalyst area, evaluation criteria.

**Introduction**

Electrochemical reactions such as hydrogen evolution reaction (HER), oxygen evolution reaction (OER), oxygen reduction reaction (ORR), and carbon dioxide reduction reaction (CO_2_RR), are playing increasingly important roles in the energy conversion devices toward sustainable energy technologies for carbon neutrality.^1-5^ For the above-mentioned electrochemical reactions, high-performance electrocatalysts are important to increase the energy conversion efficiency, and lower the cost of energy consumption.^6-10^ In this regard, scientists have developed various high-performance electrocatalysts to accelerate the reaction rate. Most reports show that the as-synthesized electrocatalysts exhibit excellent catalytic performance under their experimental conditions. Note that the experimental conditions and performance evaluation methods vary among literature, making it difficult to compare the performance of reported electrocatalysts among literature accurately.

Overpotential (*η*), the additional potential to drive the reaction to reach a specific current density, is widely used as the primary parameter to evaluate the activity of electrocatalysts.^11-13^ Usually, electrochemical measurements are carried out in a three-electrode system, and *η* at the current density of 10 mA cm^-2^ (defined as *η* @10 mA cm^-2^ hereafter) is used to describe the activity of electrocatalyst both in HER and OER.^14-15^ However, the reported *η* @10 mA cm^-2^ shows different values even for the most commonly reference catalysts, i.e., platinum (Pt) catalysts. In Fig. 1, we show *η* @10 mA cm^-2^ for HER in both acid (Fig. 1a) and alkaline (Fig. 1b) electrolytes and find that the values of *η* @10 mA cm^-2^ of Pt catalysts show a wide range from 20 mV to 100 mV, sometimes even larger than 100 mV. Though these values are all obtained by normalizing the projected electrode area (EA) of Pt catalysts, the morphologies of these Pt catalysts differ (Tables S1and S2 in the Supplementary Information), such as foil, wire, film, or mesh. In addition, the testing conditions and catalyst features are also different in these literature, such as size of electrolyzer, type and temperature of electrolyte, and loading quantity of catalysts. Note that the *η* of electrocatalyst would change not only with catalyst microstructure,^16-17^ but also with resistances like solution resistance (R*_s_*) and charge transfer resistance (R*_ct_*).^18-19^ Furthermore, the working environment such as the electrolyte temperature can also affect the thermodynamics of the electrocatalyst, resulting in a change of *η*.^20-22^ Fig. 2 summarizes several factors which may affect the performance of electrocatalysts, including intrinsic activity, resistance effect, environmental effect, loading quantity, catalyst microstructure, and evaluation method. Therefore, to maintain sound progress in the electrocatalytic ﬁeld, adopting a standard methodology for measuring and analyzing the catalytic performance of electrocatalysts is urgently needed.

**Figure 1**. **A summary of HER activity of Pt electrocatalysts reported in the literature**. The overpotentials of Pt catalysts at the current density of 10 mA cm^-2^_EA_ (a) in acid electrolyte and (b) in alkaline electrolyte. The diameters of the circles roughly represent the number of catalysts in the specified overpotential range. The Pt catalysts include foil, wire, film, and mesh, and the data are collected from the literature. Details are shown in Tables S1 and S2 in the Supplementary Information.

**Figure 2**. **The factors that will influence the evaluation of electrocatalyst performance**. These factors include the intrinsic activity, resistance effect, environmental effect, loading quantity, catalyst microstructure, and evaluation method.

Here we study the underlying reasons for the diverse performance of Pt catalysts and aim to provide evaluation criteria for reliable electrochemical measurements. We have focused on the following two aspects. The first aspect is related to the experimental measurements, including (i) the distance between reference electrode (RE) and working electrode (WE) which represents the R*_s_*, (ii) temperature of electrolyte, and (iii) loading quantity and microstructure of the catalysts. The second aspect is related to data processing, i.e., the method to determine and normalize the area of catalysts. Finally, we advocate a set of standardized testing protocols and data processing methods to evaluate the performance of electrocatalyst.

**Experimental Section**

The Pt foil (purity: 99.5%), Pt/C with different mass ratios of Pt, and Pt black were purchased from Alfa Aesar. IrO_2_ was purchased from Aladdin. The tantalum disulfides were synthesized by the chemical vapor deposition method as previously reported ^[23]^. All electrochemical measurements were conducted on the electrochemical station (VMP-300, Bio-Logic, France) with a standard three-electrode system in a 0.5 M H_2_SO_4_ electrolyte or a 1.0 M KOH electrolyte. We used a graphite rod as the counter electrode, a saturated Ag/AgCl electrode (in acidic electrolyte) or an Hg/HgO (in alkaline electrolyte) as the reference electrode, and the platinum-based catalysts as the working electrode for HER and commercial IrO_2_ for OER tests. Before the electrochemical tests, the electrolyte was purged with N_2_ gas (99.999%) for 30 min. All reported potentials mentioned hereafter were calibrated by reversible hydrogen electrode (RHE). The calibration of the saturated Ag/AgCl electrode was performed in a hydrogen saturated electrolyte made of 0.5 M H_2_SO_4_ with Pt foils as both working and counter electrodes. The calibration of the Hg/HgO electrode was performed in a hydrogen saturated electrolyte made of 1.0 KOH with Pt foils as both working and counter electrodes. Linear sweep voltammetry (LSV) test was performed at a scan rate of 0.5 mV s^−1^. The average of the two potentials at which the current crossed zero was considered to be the thermodynamic potential for the hydrogen electrode reaction. As a result, the calibration in a 0.5 M H_2_SO_4_ electrolyte was based on the following equation: E_(RHE)_ = E (Ag/AgCl) +0.0591pH + 0.205 V. The calibration in a 1.0 KOH electrolyte was based on the following equation: E_(RHE)_ = E (Hg/HgO) +0.0591pH + 0.097 V. HER activity of different samples was evaluated based on LSV with a scan rate of 2 mV s^−1^. Nyquist plots were obtained at an overpotential of 30 mV and the sweeping frequencies ranged from 1 MHz to 0.1 Hz. The electrochemical surface area (ECSA) of the catalyst was calculated based on the following equation:

ECSA_Pt_ = C_dl_ / (0.196 mF cm^-2^) Equation (1)

Where C_dl_ was the capacitance of the electrochemical double-layer, the coefficient 0.196 mF cm^-2^ was the value of the specific capacitance on polycrystalline Pt.

In addition, the hydrogen desorption area (HDA) of the catalyst was calculated based on the following equation:

HDA_Pt_ = Q_H_ / (210 µC cm^-2^) Equation (2)

Where Q_H_ was the charge for hydrogen desorption, the coefficient 210 µC cm^-2^ was the value of the hydrogen desorption charge density on polycrystalline Pt.

**Results and Discussion**

To investigate the origin of varied performance of Pt catalysts, we use the commercial Pt-based catalysts in HER tests. First, we explore the resistance effect on catalytic performance. In this case, a Pt foil (Figs. S1-S2) serves as WE in a three-electrode cell. The three-electrode cell consists of two circuits (Fig. 3a). The polarization circuit between WE and counter electrode (CE) monitors charge transfer and polarization current, and the measuring circuit between RE and WE controls the potential and measures the electrochemical reaction process of WE.^24^ A voltage drop between RE and WE would be generated due to the existence of R*_s_* and the potential can be partially corrected by performing iR_c_ compensation (Fig. 3b). The R*_s_* will increase with increasing the distance between RE and WE, thus the applied potential (between WE and CE) in reaction will decrease. The activity of Pt catalysts normalized by EA is first evaluated by varying distances between the RE and the WE (Fig. S3a). The results show that *η* @10 mA cm^-2^_EA_ of Pt catalyst increases from 58, 66, to 71 mV (all without iR_c_ compensation) when the distances between the RE and the WE enlarging from 0.2, 5.0, to 8.0 cm (Figs. 3c and S3b, in a 0.5 M H_2_SO_4_ electrolyte). Electrochemical impedance spectroscopy (EIS) results show that the R*_s_* also increases from 0.3, 0.9, to 1.3 Ω under these three conditions (Fig. 3c). It shows similar results in a 1.0 M KOH electrolyte, where *η* @10 mA cm^-2^_EA_ of Pt catalyst increases from 63, 68, to 72 mV (all without iR_c_ compensation) and the R*_s_* also increases from 0.3, 0.8, to 1.2 Ω when the distances between the WE and the RE enlarging from 0.2, 5.0, to 8.0 cm (Figs. S3c-d). Such increases of overpotential when the distances between RE and WE are enlarging due to extra voltage drop caused by the increase of R*_s_*. Although we calculate the *η* @10 mA cm^-2^_EA_ with iR_c_ compensations (85%) (Fig. 3d), the voltage loss remains. It is hard to eliminate the resistance effect completely because the uncompensated resistance (R_u_) still remains in the electrochemical cell. Resistance effects are common in the electrochemical system, regardless of catalytic materials or reactions. Besides Pt foil for HER, we also investigate the resistance effect on the activity of different catalyst materials (*e.g.*, tantalum disulfides for HER, Fig. S4) and reactions (*e.g.*, iridium dioxides for OER, Fig. S5). Both cases show that the *η* @10 mA cm^-2^_EA_ of catalyst and the R*_s_* increase when the distances between RE and WE enlarging from 0.2 cm to 8.0 cm. These results reconfirm the influence of resistance effect on the performance of electrocatalysts. The actual activity of the catalyst can be measured accurately only by minimizing the resistance effect as small as possible.

**Figure 3**. **Catalytic activity of Pt foil *vs.* solution resistance**. (a) Schematic of a three-electrode electrochemical system. (b) Schematic of a three-electrode cell with different R*_s_* tuned by the distances between RE and WE. R*_s_* represents solution resistance, R_c_ represents compensated resistance, and R_u_ represents uncompensated resistance. (c) The overpotentials of Pt foil at the current density of 10 mA cm^-2^_EA_ and R*_s_* at different distances between RE and WE in a 0.5 M H_2_SO_4_ electrolyte. (d) The overpotentials of Pt foil at the current density of 10 mA cm^-2^_EA_ with or without the iR_c_ compensation (85%).

Next, we study the environmental effect on the catalytic performance of catalysts by changing the electrolyte temperature. Three electrochemical systems are designed with different electrolyte temperatures (5, 25, and 60 ℃) to investigate the HER activity of Pt foil catalyst. We find that the *η* gradually decreases as electrolyte temperature increases (Figs. 4a and S6). The corresponding Tafel slopes also decrease from 40, 37, to 33 mV dec^-1^ as electrolyte temperatures increase from 5, 25, to 60 ℃ (Fig. 4b). These results are consistent with the Arrhenius equation$k=Ae^{\frac{-E_{a}}{RT}}$, where *k* the rate constant of the reaction, A the pre-exponential factor, E_a_ the activation energy, and T the temperature. Note that the electrolyte temperature may increase significantly due to the heat effect caused by ohmic heating under large-current-density operation. Our results show that the electrolyte temperature does not change obviously when the operating current density is smaller than 100 mA cm^-2^, while it increases sharply when the operating current density is larger than 500 mA cm^-2^ (Fig. S7). For example, the electrolyte temperature increases from 25.0 ℃ to 69.1 ℃ at 2,000 mA cm^-2^_EA_ after 5 hours operation. Therefore, a loss of performance may be offset by heat effect during the stability test at large current densities and cause a false-positive result. Here we investigate the heat effect on the electrocatalyst stability by controlling the electrolyte temperature with or without electrolyte circulation. As shown in Fig. 4c, the electrolyte temperature gradually increases during the stability testing at 1,000 mA cm^-2^_EA_ in the case without electrolyte circulation, while the temperature remains the same when we use the circulated electrolyte. As a result, the current density shows negligible decay which is incorrect after 5 h stability test without circulating electrolyte, while it decreases by 18% which is correct after 5 h stability test with electrolyte circulation. The degradation of the catalyst performance is due to the protonation of active sites on Pt surface when the reaction continues (Fig. 4d). The difference between two cases with and without electrolyte circulation is because the thermodynamics of electrocatalyst will improve when the working temperature increases due to the heat effect, which would compensate the decrease of current density over long-term operation. This phenomenon usually causes a misleading in evaluating electrocatalyst stability especially under large-current-density operation. Therefore, care must be taken to eliminate temperature effect when evaluating the large-current-density performance of electrocatalysts.

**Figure 4**. **The effect of electrolyte temperature on catalytic performance**. (a) The overpotentials of Pt foil catalysts at current density of 10 mA cm^-2^_EA_ at different electrolyte temperatures of 5, 25, and 60 ℃ in a 0.5 M H_2_SO_4_ electrolyte. (b) The Tafel slopes of Pt foil catalysts at different electrolyte temperatures. (c) The electrolyte temperature versus time with and without electrolyte circulation tested at 1,000 mA cm^-2^_EA_. (d) The i-t curves of Pt foil catalysts with and without electrolyte circulation.

Then we investigate the effects of loading quantity and microstructure of Pt catalysts on their performance. Regarding the noble metal catalysts such as Pt, different mass ratios of Pt (mass ratios usually are 10%, 20%, and 40%) in commercial Pt/C catalysts also have been used to compare the catalytic activity in literature. We have conducted the electrochemical tests based on three different mass ratios of Pt under the same conditions and normalized their activities by both projected EA and quantity, respectively (Fig. S8). The results show that the current density calculated by EA increases from 24, 35, to 44 mA cm^-2^ when increasing the mass ratios of Pt in Pt/C catalysts from 10%, 20%, to 40% at the *η* of 50 mV. As a contrast, the current density calculated by quantity of Pt decreases from 596, 437, to 320 mA mg^-1^ when increasing the mass ratios of Pt in Pt/C catalysts from 10%, 20%, to 40% (Fig. 5a). This result indicates that for the same catalyst, opposite trend of catalytic performance can be generated from different evaluation methods. In addition, even for current normalized by EA, we find that this EA-normalized current differs with electrode sizes (Figs. 5b and S9). Because electrodes with different sizes (i.e., different EA) may cause different degrees of uneven distribution of electric fields, polarization, and R*_s_*, resulting in different reaction dynamics of local active sites on catalysts. To avoid the above effects, we recommend that the EA of CE should be twice larger than that of WE in a three-electrode electrochemical cell. We also find that the catalytic performance shows significant differences even though the electrodes have the same geometric EA. Two catalysts are used here including Pt black (Pt nanoparticles loaded on Pt foil) and Pt foil. As shown in Fig. 5c, Pt black shows a porous structure both from the top view and the cross-sectional view while Pt foil is flat. Therefore, Pt black has a much larger surface area than that of flat Pt foil, indicating large numbers of active sites can be exposed in the electrolyte for the former case. The catalytic activities of Pt black and Pt foil have been investigated based on different kinds of normalized areas including EA, ECSA (calculated by equation 1), and HDA (calculated by equation 2) (Figs. 5d and S10). The results show that Pt black only needs a *η* of 31 mV to reach 10 mA cm^-2^ normalized by EA, which is smaller than that of Pt foil (57 mV). However, the *η* of Pt black at 10 mA cm^-2^ normalized by ECSA and HDA are 189 and 192 mV, respectively, and are larger than that of Pt foil (135 mV and 37 mV). There are two possible reasons accounting for these results. One is that the surface average coordination numbers of Pt black is lower than that of Pt foil, resulting in the worse activity of Pt black than Pt foil. Another is that the proportion of active sites involved in the HER of Pt black is smaller than that of Pt foil due to limited gas diffusion and mass transfer in Pt black. Therefore, the evaluation of catalytic activity differs when using different methods to normalize catalyst areas.

**Figure 5**. **Catalytic activity of Pt catalysts based on the EA, loading quantity, size, and different methods to normalize catalyst areas.** (a) The current density of different mass ratios of Pt (10%, 20%, and 40%) of Pt/C catalysts in a 0.5 M H_2_SO_4_ electrolyte calculated by EA and loading quantity, respectively. The loading quantity of Pt is 8 µg, 16 µg, and 32 µg, respectively. (b) The measured and EA-normalized current densities of Pt catalysts at a constant overpotential of 58 mV. The Pt catalysts with different sizes (i.e., EA) are used during experiments. (c) The SEM images of Pt foil (left panel) and Pt black (right panel) from top view and cross-sectional view, respectively. (d) The overpotentials of Pt foil and Pt black at 10 mA cm^-2^ with different methods to normalize catalyst areas, including EA, ECSA, and HDA.

**Conclusion and Outlook**

We have discussed several factors that affect the catalytic performance of electrocatalysts. We find that the performance of Pt electrocatalysts varies by using different experimental conditions and evaluation methods, including resistance effect, electrolyte temperature, loading quantity, microstructure of catalysts, and the methods to normalize the catalyst area. To evaluate the performance of electrocatalysts more accurately and reliably, we advocate the establishment of testing and evaluation criteria. The following are some proposed guides for accurately evaluating the performance of electrocatalysts.

1. **Provide a checklist for experimental details and evaluation methods**. The detailed measurement conditions and evaluation method of electrocatalysts should be described in detail in papers (Table 1). First, to fairly compare the testing results and evaluate the practicality of the electrocatalyst, the electrochemical cell should keep the same conditions during the testing process and be consistent with industrial standards as much as possible. Second, criteria such as environmental temperature, pH value, pressure, testing parameters, and evaluation method should be offered in detail as much as possible in the electrocatalytic performance statement. Third, the reasonable use of RE and CE is also critical for performance evaluation. We recommend use the saturated calomel electrode or hydrogen electrode as RE in acidic electrolyte, and use mercuric oxide electrode as RE in alkaline electrolyte. In addition, platinum- and graphite-based electrodes are not suitable to serve as OER electrode in acidic electrolyte, because the dissolved matter from CE may migrate to the surface of WE and affect the catalytic activity of WE.
2. **Minimize the resistance effect on performance evaluation.** The resistance effect is trouble in the electrolysis testing, which would make the measurement of potential inaccurate. We suggest that researchers can shorten the distance between RE and WE in an electrochemical cell to lower the R*_s_*, and strongly recommend lowering the R*_s_* by using the Luggin-Haber capillary. In addition, iR compensation is also needed to eliminate the resistance effect of the electrolyte on activity evaluation. Furthermore, the interference of thermal effect should be avoided by using circulated electrolyte, which is necessary especially when operating at large current density over long time.
3. **Set** **normalization standards**. The selection of catalyst area for normalization of current density is generally depended on the type and microstructure of catalysts. Regarding non-noble metal electrocatalysts, we recommend use EA to normalize the current density for flat catalysts, and use ECSA or HDA to normalize current density for porous catalysts. For noble metal electrocatalysts, the amount of noble metal catalysts is of concern because it shares a noticeable percentage of cost in the energy conversion system. In this case, the mass activity should be used and is generally suitable for noble metal electrocatalysts due to the high cost and scarcity.
4. **Define performance evaluation method**. The evaluation methods of catalytic performance vary due to the different types of catalytic materials and different purposes. In general, the evaluation methods can be divided into two categories. One is the evaluation of intrinsic catalytic activity and another one is the evaluation of overall catalyst performance. For the former case focusing on fundamental studies, it is indispensable to calculate the numbers of active sites. For the latter case that is more application-orientated, we should consider the loading quantity and cost of catalyst, especially when using a large amount of noble metal catalysts.

Nothing can be accomplished without standards. Establishing a standardized evaluation system of catalytic activity is a prerequisite for the rapid development and large-scale implementation of the electrocatalytic energy conversion technologies. Meanwhile, it is essential to identify the atomic structure of electrocatalysts and active sites with theoretical and in-situ experimental approaches for understanding the catalyst activity. We believe that on the above basis, the advances of new catalytic materials can be evaluated accurately and reliably.

**Table 1. A checklist for the evaluation criteria of electrocatalytic performance**

**Declaration of Competing Interest**

The authors declare no competing financial interest.

**Acknowledgements**

The authors thank Ms. Fengning Yang for the fruitful discussions. We acknowledge financial support from the Opening Project of the Key Laboratory of Two-Dimensional Materials (Hunan Province), Hunan University (KF20200002), the National Science Fund for Distinguished Young Scholars (No. 52125309), the Guangdong Innovative and Entrepreneurial Research Team Program (No. 2017ZT07C341), and the Shenzhen Basic Research Project (No. JCYJ20200109144620815).

**References**

1. Mallapaty, S., How China Could Be Carbon Neutral by Mid-Century. Nature 586 (7830) (2020) 482-483.

2. Electrocatalysis for the Generation and Consumption of Fuels. Nat. Rev. Chem. 2 (4) (2018) 0125.

3. Montoya, J. H.; Seitz, L. C.; Chakthranont, P.; Vojvodic, A.; Jaramillo, T. F.; Norskov, J. K., Materials for Solar Fuels and Chemicals. Nat. Mater. 16(1) (2017) 70-81.

4. Zhang, C.; Luo, Y. T.; Tan, J. Y.; Yu, Q. M.; Yang, F. N.; Zhang, Z. Y.; Yang, L. S.; Cheng, H. M.; Liu, B. L., High-Throughput Production of Cheap Mineral-Based Two-Dimensional Electrocatalysts for High-Current-Density Hydrogen Evolution. Nat. Commun. 11 (2020) 3724.

5. Yu, Q. M.; Zhang, Z. Y.; Qiu, S. Y.; Luo, Y. T.; Liu, Z. B.; Yang, F. N.; Liu, H. M.; Ge, S. Y.; Zou, X. L.; Ding, B. F.; Ren, W. C.; Cheng, H. M.; Sun, C. H.; Liu, B. L., A Ta-TaS_2_ Monolith Catalyst with Robust and Metallic Interface for Superior Hydrogen Evolution. Nat. Commun. 12 (2021) 6051.

6. Seh, Z. W.; Kibsgaard, J.; Dickens, C. F.; Chorkendorff, I. B.; Norskov, J. K.; Jaramillo, T. F., Combining Theory and Experiment in Electrocatalysis: Insights into Materials Design. Science *355*(6321) (2017) aad4998.

7. Roger, I.; Shipman, M. A.; Symes, M. D., Earth-Abundant Catalysts for Electrochemical and Photoelectrochemical Water Splitting. Nat. Rev. Chem. 1 (1) (2017) 0003.

8. Hong, W. T.; Risch, M.; Stoerzinger, K. A.; Grimaud, A.; Suntivich, J.; Shao-Horn, Y., Toward the Rational Design of Non-Precious Transition Metal Oxides for Oxygen Electrocatalysis. Energ. Environ. Sci*.* 8 (5) (2015) 1404-1427.

9. Luo, Y. T.; Tang, L.; Khan, U.; Yu, Q. M.; Cheng, H. M.; Zou, X. L.; Liu, B. L., Morphology and Surface Chemistry Engineering for pH-Universal Catalysts toward Hydrogen Evolution at Large Current Density. Nat. Commun. 10 (2019) 269.

10. Shi, Q. R.; Zhu, C. Z.; Du, D.; Lin, Y. H., Robust Noble Metal-Based Electrocatalysts for Oxygen Evolution Reaction. Chem. Soc. Rev*.* 48 (12) (2019) 3181-3192.

11. Yu, W. T.; Porosoff, M. D.; Chen, J. G. G., Review of Pt-Based Bimetallic Catalysis: From Model Surfaces to Supported Catalysts. Chem. Rev. 112 (11) (2012) 5780-5817.

12. Huo, W.-Y.; Wang, S.-Q.; Zhu, W.-H.; Zhang, Z.-L.; Fang, F.; Xie, Z.-H.; Jiang, J.-Q., Recent Progress on High-Entropy Materials for Electrocatalytic Water Splitting Applications. Tungsten 3 (2) (2021) 161-180.

13. Yu, Q. M.; Luo, Y. T.; Mahmood, A.; Liu, B. L.; Cheng, H. M., Engineering Two-Dimensional Materials and Their Heterostructures as High-Performance Electrocatalysts. Electrochem. Energ. Rev*. 2* (3) (2019) 373-394.

14. Wei, C.; Xu, Z. C. J., The Comprehensive Understanding of 10 mA cm(geo)(-2) as an Evaluation Parameter for Electrochemical Water Splitting. Small Methods 2 (11) (2018) 1800168.

15. Wei, C.; Rao, R. R.; Peng, J. Y.; Huang, B. T.; Stephens, I. E. L.; Risch, M.; Xu, Z. C. J.; Shao-Horn, Y., Recommended Practices and Benchmark Activity for Hydrogen and Oxygen Electrocatalysis in Water Splitting and Fuel Cells. Adv. Mater. 31 (31) (2019**)** 1806296.

16. Voiry, D.; Chhowalla, M.; Gogotsi, Y.; Kotov, N. A.; Li, Y.; Penner, R. M.; Schaak, R. E.; Weiss, P. S., Best Practices for Reporting Electrocatalytic Performance of Nanomaterials. ACS Nano 12 (10) (2018) 9635-9638.

17. Sun, S. N.; Li, H. Y.; Xu, Z. C. J., Impact of Surface Area in Evaluation of Catalyst Activity. Joule 2 (6) (2018**)** 1024-1027.

18. Van der Vliet, D.; Strmcnik, D. S.; Wang, C.; Stamenkovic, V. R.; Markovic, N. M.; Koper, M. T. M., On the Importance of Correcting for the Uncompensated Ohmic Resistance in Model Experiments of the Oxygen Reduction Reaction. J. Electroanal. Chem. 666 (2012) 89-89.

19. Voiry, D.; Fullon, R.; Yang, J. E.; Silva, C. D. C. E.; Kappera, R.; Bozkurt, I.; Kaplan, D.; Lagos, M. J.; Batson, P. E.; Gupta, G.; Mohite, A. D.; Dong, L.; Er, D. Q.; Shenoy, V. B.; Asefa, T.; Chhowalla, M., The Role of Electronic Coupling between Substrate and 2D MoS_2_ Nanosheets in Electrocatalytic Production of Hydrogen. Nat. Mater. 15 (9) (2016) 1003-1009.

20. Alia, S. M.; Pivovar, B. S., Evaluating Hydrogen Evolution and Oxidation in Alkaline Media to Establish Baselines. J. Electrochem. Soc. 165 (7) (2018) F441-F455.

21. Chen, J. G.; Jones, C. W.; Linic, S.; Stamenkovic, V. R., Best Practices in Pursuit of Topics in Heterogeneous Electrocatalysis. ACS Catal. 7 (9) (2017) 6392-6393.

22. Niu, S. Q.; Li, S. W.; Du, Y. C.; Han, X. J.; Xu, P., How to Reliably Report the Overpotential of an Electrocatalyst. ACS Energ. Lett. 5 (4) (2020) 1083-1087.

23. Yu, Q. M.; Luo, Y. T.; Qiu, S. Y.; Li, Q. Y.; Cai, Z. Y.; Zhang, Z. Y.; Liu, J. M.; Sun, C. H.; Liu, B. L.; Tuning The Hydrogen Evolution Performance of Metallic 2D Tantalum Disulfide by Interfacial Engineering. ACS Nano 13 (10) (2019) 11874-11881.

24. Bard, A. J.; Faulkner, L. R. In., Electrochemical Methods: Fundamentals and Applications, 1980.
